# Patient perspectives on electronic patient-reported outcome-based symptom management after lung cancer surgery: a qualitative study

**DOI:** 10.1186/s41687-026-01083-4

**Published:** 2026-05-19

**Authors:** Yadi Zhang, Xin Gao, Cheng Lei, Hongfan Yu, Yangjun Liu, Xin Shelley Wang, Cecilia Pompili, Yang Pu, Wei Xu, Yaqin Wang, Jia Liao, Xing Wei, Fang Liu, Qiang Li, Qiuling Shi, Wei Dai, Linlin Fan

**Affiliations:** 1 Department of Thoracic Surgery, Sichuan Clinical Research Center for Cancer, Sichuan Cancer Hospital & Institute, Sichuan Cancer Center, University of Electronic Science and Technology of China, No. 55, Section 4, South Renmin Road, Chengdu, Sichuan 610041 China; 2 State Key Laboratory of Ultrasound in Medicine and Engineering, College of Biomedical Engineering, Chongqing Medical University, Chongqing, China; 3 Department of Medical Epidemiology and Biostatistics, Karolinska Institutet, Stockholm, Sweden; 4 Department of Symptom Research, The University of Texas MD Anderson Cancer Center, Houston, TX USA; 5 Department of Thoracic Surgery, Institute for Clinical & Applied Health Research, University of Hull, Hull, UK; 6 Section of Patient Centred Outcomes Research, Leeds Institute of Medical Research at St James’s, University of Leeds, Leeds, UK; 7 Chongqing College of Traditional Chinese Medicine, Chongqing, China; 8 College of Public Health, Chongqing Medical University, Chongqing, China; 9 Department of Integrated Traditional Chinese and Western Medicine, Sichuan Clinical Research Center for Cancer, Sichuan Cancer Hospital & Institute, Sichuan Cancer Center, University of Electronic Science and Technology of China, Chengdu, Sichuan China

**Keywords:** Electronic patient-reported outcome, Symptom monitoring, Lung cancer surgery, Qualitative study

## Abstract

**Background:**

Patients frequently experience severe symptoms after lung cancer surgery. Symptom management using electronic patient-reported outcomes (ePRO) can improve postoperative symptom burden, enhance functional status, and reduce complications. This qualitative study aimed to explore patient perspectives on ePRO-based symptom management.

**Methodology:**

Patients in a multicentre, randomised controlled trial (CN-PRO-Lung 2) conducted in China were asked to participate in qualitative interviews. In the trial, patients with lung cancer were randomly assigned in a 1:1 ratio to receive postoperative symptom management based on ePRO or usual care. To explore perspectives on the model, semi-structured interviews were conducted with 20 patients in the ePRO group. Data were analysed using descriptive and thematic analyses.

**Results:**

The median age of patients was 53.5 years (range: 28–73 years), with 55% being female. Two major themes emerged from the qualitative interviews: patient acceptance and patient-derived recommendations. Patient acceptance included four subthemes: patient satisfaction, daily life disruption and burden, postoperative recovery assistance, and long-term implementation needs. Patients were highly satisfied with the ePRO model and felt it did not increase their burden. They also found it beneficial for recovery and recommended long-term implementation. Patient-derived recommendations included two subthemes: enhancing the ePRO model and improving the system. Patients emphasised the importance of timely doctor feedback, a user-friendly interface, recognisable app icons, and additional self-reporting sections to increase engagement and efficiency.

**Conclusions:**

ePRO-based symptom management seems to be acceptable for patients who underwent lung cancer surgery. Future research should focus on optimising the ePRO model and system to better meet patient needs and facilitate its clinical implementation.

**Supplementary Information:**

The online version contains supplementary material available at 10.1186/s41687-026-01083-4.

## Introduction

Lung cancer remains the leading cause of cancer-related mortality and incidence globally, with new cases in China accounting for approximately 40% of those recorded worldwide annually [1, 2]. Surgery is the cornerstone of curative treatment for early-stage lung cancer [2–4], and the number of lung cancer surgeries performed annually in China is currently the highest globally [2–4]. However, surgical procedures are associated with significant physical and psychosocial trauma, and many patients experience severe symptoms (e.g. pain, cough, distress) for an extended period after surgery and discharge [5–7].

Patient-centred care models highlight proactive symptom monitoring to optimise recovery throughout the disease course [8–10]. In our previous multicentre, randomised controlled trial (CN-PRO-Lung 2), postoperative patients with lung cancer managed via electronic patient-reported outcome (ePRO)-based symptom management demonstrated better symptom control at 1 month after discharge than did those who received usual care [11], with sustained benefits observed at the 1-year follow-up [12]. Despite these benefits, the implementation of ePRO-based care faces barriers from multiple stakeholders, including patients (e.g. lack of digital literacy), clinicians (e.g. increased workload), and institutions (e.g. lack of electronic infrastructure).

A qualitative study regarding implementation of ePRO symptom monitoring after thoracic surgery in the United States identified several patient-perceived barriers, such as limited physical and digital access and unclear system purpose, and facilitators, such as engagement with the surgical care team regarding ePRO use [13]. However, no similar research has explored patient perspectives in China, where variations in the healthcare system and cultural context might influence the adoption of ePRO-based postoperative care. Thus, this qualitative study aimed to investigate patient perceptions and experiences on the ePRO model following lung cancer surgery in China.

## Methods

### Study design and patients

Patients were purposively sampled from a multicentre, randomised controlled trial (CN-PRO-Lung 2) conducted in China [11, 12, 14]. The aim of the trial was to evaluate the efficacy of ePRO-based symptom management in postoperative patients with lung cancer. Purposeful sampling involves intentional selection of participants who can provide rich and diverse insights into the research question [15]. We employed maximum variation sampling to capture a comprehensive range of perspectives on the ePRO model. The operational criteria for variation were based on core factors hypothesized to influence digital health engagement, including age, education level, socioeconomic status, and clinical characteristics. The selection process was iterative: as recruitment progressed, we continuously monitored the diversity of the sample. To avoid ‘homogeneity bias,’ we deliberately sought out participants who were underrepresented in earlier stages (those with lower household income or from rural centres). This ensured that the findings reflect the experiences of a heterogeneous group of patients, ranging from those with high digital proficiency to those facing potential barriers to ePRO use. Because this qualitative study focused on patient experiences with the ePRO model, only participants from the intervention group were interviewed; it was not intended to compare experiences between trial arms. Participants from three centres formed the interview group, in proportion to the total number of enrolled patients from each centre. Patients were recruited during the period from the time of discharge to 1 year after discharge. Thus, the interview sample was intended to reflect diversity of experience rather than statistical representativeness.

This study was approved by the institutional review board of Sichuan Cancer Hospital (approval number: SCCHEC-02-2018-045; date of approval: November 22, 2018) and conducted according to the tenets of the Declaration of Helsinki. All patients signed a written informed consent form. Participation was voluntary, with no financial compensation, but participants were offered free long-term medical consultation as a form of non-financial support. This descriptive qualitative study was reported in accordance with the Consolidated Criteria for Reporting Qualitative Research (COREQ) checklist [16].

### ePRO intervention trial

**Fig. 1:**
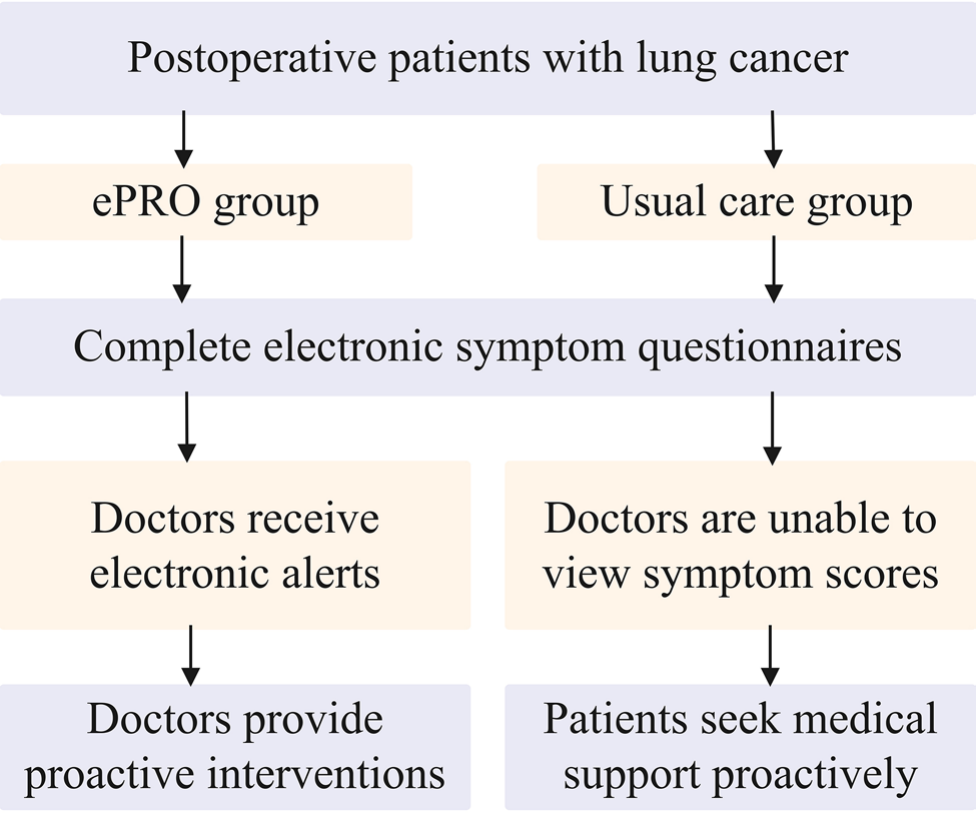
Schematic diagram of ePRO intervention and usual care. *ePRO* electronic patient-reported outcomes

A total of 166 patients were included in the trial and divided into an intervention group (ePRO group) and a control group (usual care group) [11]. Patients in the intervention group received ePRO-based symptom management after surgery. Patients completed the MDASI-LC, which assesses 16 and 6 symptom-severity and symptom-interference items, respectively. Severity was rated on a 0–10 scale (0 = ‘no symptom’; 10 = ‘worst imaginable’), and interference items were rated using the same anchors (0 = ‘did not interfere’; 10 = ‘interfered completely’). This approach captured symptom occurrence, severity, and their impact on daily functioning [17]. The surgeons were required to address the alert within 24 h, including answering inquiries, providing patient education, prescribing medications, and offering clinic visit recommendations. Alerts were handled by the on-duty surgeon during the weekends and off-hours. This process constituted the closed-loop ePRO symptom management model, referring to the overall care process of symptom monitoring, real-time alerting, and early intervention to ensure timely care [14]. The ePRO system, by contrast, referred to the electronic platform through which patients submitted symptom data and the clinical team managed those data. After submission, patients saw only a brief confirmation message (“Thank you for your participation”) and were not informed of any symptom results. Patients in the control group received usual postoperative care. They completed the same electronic symptom questionnaires completed by those in the intervention group; however, their responses did not generate alerts, and the clinical team could not access the reported scores. Symptom management was based on routine morning and afternoon ward rounds during hospitalisation, or initiated by patients proactively contacting their attending physicians to report symptoms. After discharge, patients could obtain medical support only if they proactively contacted the clinical team or sought care at a local healthcare facility (Fig. 1).

Symptom and quality-of-life assessments were conducted using two validated instruments: the MD Anderson Symptom Inventory–Lung Cancer module [18], which includes 16 symptom items and 6 interference items for the evaluation of lung cancer-specific symptom burden and its impact on daily functioning, and a single-item global quality-of-life scale [19]. Patients completed the questionnaires in a WeChat mini-program, which is a lightweight application embedded within WeChat. They accessed it on their own smartphones through password-protected accounts. Submitted data were transmitted to the hospital’s REDCap server. The average time to complete the questionnaires was approximately 3–5 min. Assessments were conducted once before surgery, daily after surgery for up to 14 days, twice weekly thereafter for up to 4 weeks, and once at 3, 6, 9, and 12 months after discharge [12].

### Interview guide

After three rounds of discussions and revisions among our team involving thoracic surgeons, nurses, and methodology experts specialised in ePRO design and implementation, an interview guide containing seven questions was developed (see supplementary text in Additional File 1) [20, 21]. This interview guide outlined the background and objectives of the survey and explained the confidentiality of the interview after obtaining patient consent. The main body of our interview consisted of two sections: (1) overall experiences and feelings with the ePRO model and system and (2) recommendations for improving the ePRO model and system, including prompts to explore dissatisfaction, perceived burden, interference with daily life, and suggestions for improvement, with follow-up probing used when needed.

### Data collection

The primary outcome measures of the trial have been reported [11], indicating that ePRO-based symptom management after lung cancer surgery reduced symptom burden, improved functional status, and reduced complications. This qualitative component complemented the parent trial by providing patient perspectives that helped interpret the quantitative findings and identify implementation needs for future optimisation of the ePRO-based symptom management model. The qualitative interviews in this article represent a secondary outcome of the main study. We employed a qualitative research method to explore the acceptance and burden among patients in the intervention group [22].

The sample size was determined according to the principle of data saturation, and a total of 20 patients were ultimately invited for qualitative interviews [23]. Sample size determination was guided by the concept of data saturation, defined as the point at which no new themes or codes appeared in two successive interviews [24]. Following interviews with 18 participants, we conducted two further interviews, which likewise revealed no additional insights. Consequently, 20 patients were included in the qualitative study. All 20 patients approached for interviews provided consent, and no refusals were recorded. Trained researchers conducted one-on-one, semi-structured telephone interviews with each participant; each interview lasted 10–30 min, and additional probing questions were asked when required. Although the interviews were relatively brief, the interview guide was focused on patients’ experiences with the ePRO model, and follow-up probing questions were used to elicit further detail when needed. Subsequently, the audio-recorded interviews were uploaded to a third-party service provider within 48 h and transcribed verbatim. The transcripts were manually cross-checked by two researchers to ensure accuracy. The qualitative interviews with the 20 patients were conducted between 21 July 2020 and 2 March 2021.

### Data analysis

Data collection and analysis were iteratively conducted. Interviews were conducted in Mandarin and audio-recorded. Recordings were transcribed verbatim in Chinese; textual data were managed in NVivo 12 (QSR International, 2020) and analysed using thematic analysis on the original Chinese transcripts. To ensure the rigor and trustworthiness of the analysis, we used the following strategies: (1) Investigator triangulation: Two researchers independently coded a subset of transcripts, with discrepancies resolved through discussion and, if necessary, arbitration by a third researcher to ensure coding consistency. (2) An iterative process: A preliminary codebook was developed and refined throughout the analysis to enhance its fit with the data. (3) Member checking: Preliminary findings were shared with some participants to validate the interpretations. (4) Audit trail: A detailed record of analytical decisions was maintained. (5) Thick description: Detailed characteristics of the study context and participants are provided to facilitate transferability judgments.

## Results

### Baseline demographic characteristics

Overall, 20/83 (24.1%) participants from the ePRO group participated in the interviews. The median age was 53.5 years (range: 28–73 years); 55% were female and 50% had an education beyond high school. Additionally, 50% of patients were employed full time, whereas 50% were retired or unemployed (Table 1). The median time from alert to physician intervention was 39.5 min (range: 1–946 min).

**Table 1:** Patient characteristics (*n* = 20)

Characteristic	*N*	%
Age (years), median (range)	53.5 (28–73)	
Age (years), mean (SD)	55.3 (11.3)	
Age		
≤ 54	10	50
> 54	10	50
Sex		
Male	9	45
Female	11	55
Education level		
≤High school	10	50
> High school	10	50
Employment status		
Full time	10	50
Retired or unemployed	10	50
Marital status		
Single	1	5
Married	19	95
Annual household income		
< 100,000 RMB	8	40
≥ 100,000 RMB	12	60
Lung cancer stage		
0-IA	13	65
IB-IIIA	7	35
Type of participating centre		
Cancer hospital	18	90
General hospital	2	10

### Themes

Two major themes were identified from the qualitative interviews. The first one was patient acceptance. This theme comprehensively reflected the overall attitude and value judgment of the participants towards the ePRO-based symptom management mode, covering multidimensional evaluations of parameters ranging from satisfaction and perception of effectiveness to the necessity of long-term implementation. The second theme was patient-derived recommendations. The participants put forward potential improvement recommendations in terms of management and technology. The former focused on improvement of the timeliness and interactivity of service processes, while the latter emphasised the ease of use and expansion of functions of the technical platform. Table 2 details the major themes, subthemes, and exemplary quotes.

**Table 2:** Themes related to the qualitative interviews

Theme	Subtheme	Exemplary quote
Patient acceptance	Patient satisfaction	Some symptoms didn’t apply to me, but I think this approach is acceptable. If, as you mentioned today, you genuinely review each case individually based on our submitted information to provide tailored responses, it could yield positive outcomes. The satisfaction level is quite good. (59 M)
		This definitely deserves a perfect 10/10 score. (42 F)
		Satisfied, it’s good. Quite convenient and user-friendly. (73 M)
		I personally am highly satisfied. The interface flows smoothly and is understandable for most users. (46 F)
		I find the digital form submission excellent - convenient and conducive to thoughtful responses. It avoids the pressure of feeling like taking an exam when completing forms onsite. (66 F)
	Daily life disruption and burden	I feel a bit of psychological burden because when I see you calling me, I get nervous and start to think about whether something is wrong or if something might happen. However, it doesn’t really interfere with my life. But I still feel nervous; I still feel anxious. (51 F)
		It’s not troublesome at all; it can be resolved in just a few minutes. (53 F)
		It doesn’t add any burden. For patients like us, this approach is very helpful and well-designed. (61 M)
		There’s no burden at all; your constant contact makes me feel much happier. (54 F)
	Postoperative recovery assistance	It provides some psychological reassurance. (51 F)
		This method is definitely helpful. At that time, I just felt short of breath, and the doctor advised me to use oxygen. Now I don’t have those symptoms anymore; I’ve recovered well. (47 F)
		I believe that your approach is definitely very good. Nowadays, there is a strong emphasis on postoperative rehabilitation, which I think is beneficial for recovery. Furthermore, rehabilitation is not just about the psychological support you provide; it may also include psychological and emotional support. I believe that this comprehensive approach should be helpful. (66 F)
		It’s beneficial and certainly a good thing. Having communication like this is helpful for the patients themselves and makes them feel more at ease. (61 M)
		It’s a form of psychological support. Knowing someone cares makes a difference. (52 F)
	Long-term implementation needs	I believe it is still necessary to promote this, because as patients, there is often a sense of panic. This illness is not like others where you can just treat it and be done; it raises concerns about what might happen in the future, which can lead to anxiety. (51 F)
		It’s essential for patients like me who live far away and need long-term care. This avoids unnecessary travel. (48 F)
		This model is necessary because it helps avoid the back-and-forth hassle. (42 F)
		It should definitely be expanded and maintained long-term. (46 F)
		This is crucial. Previously, after discharge, we felt neglected. Having continued support through this system is much better. (61 M)
		Yes, I hope this becomes a permanent program. (57 M)
Patient-derived recommendations	Enhancing the ePRO model	But if we complete the forms and receive no timely suggestions from doctors, patients may lose motivation to continue. Feedback needs to be prompt—delays could undermine confidence in the process. (59 M)
		I recommend that this model seems to be quite standardised, where you ask and we answer. If possible, it would be better to have an aspect for patients to discuss or share their experiences and feedback about their condition. This could enhance the process. (73 M)
		In my view, the new model should include a bit more content or perhaps provide more guidance for patients in the future. After all, the current approach heavily relies on their subjective experiences regarding their own situations. (66 F)
	Improving the ePRO system	Adding a free-text section would be ideal. If my symptoms aren’t listed, I could use a comment box to explain my situation. (72 M)
		Is the interface only horizontal? That can be improved. Also, it seems that the size cannot be adjusted. (28 M)
		I always have trouble finding that location. It took me a while to finally locate it and fill it out for you. The labelling is not very clear, so improvements are needed. (48 M)
		Yes, there should be more open-ended questions—like fill-in-the-blank fields. (47 F)
		Create a section for patients to provide additional feedback about their condition, so doctors can address all concerns at once. (73 M)
		The signature step is always problematic, maybe due to slow internet. This needs fixing. (61 M)
		At our age, we are not very familiar with smartphones, and sometimes we struggle to enter information. I recommend simplifying the verification code. (57 M)

Note: Quotes are followed by age and sex (F: female; M: male)

#### Theme 1: Patient acceptance

1. Patient satisfaction: The patients reported a high level of satisfaction with this model, and none mentioned dissatisfaction. The symptom reporting method through the electronic mini-program on personal electronic devices was highly recognised. A 46-year-old woman stated the following: ‘I personally am highly satisfied. The interface flows smoothly and is understandable for most users’.
2. Daily life disruption and burden: Almost all patients (19 out of 20) indicated that this model did not interfere with their lives or increase their burden. Only one patient expressed a slight psychological burden but did not feel that the model interfered with normal life. A 61-year-old man stated the following: ‘It doesn’t add any burden. For patients like us, this approach is very helpful and well-designed’.
3. Postoperative recovery assistance: All patients stated that ePRO-based symptom management was beneficial for postoperative recovery. Some reported that the system facilitated timely interventions that helped alleviate physical symptoms, for example, a 47-year-old woman stated the following: ‘At that time, I just felt shortness of breath, and the doctor advised me to use oxygen. Now, I don’t have those symptoms anymore; I’ve recovered well’, while others emphasised psychological reassurance and an improved sense of control, for example, a 52-year-old woman stated the following: ‘It’s a form of psychological support. Knowing someone cares (about me) makes a difference’.
4. Long-term implementation needs: All patients stated that implementation of ePRO-based symptom management is necessary as they wanted longer care from healthcare providers after discharge. Many patients noted that this management model reduced the inconveniences of repeated visits during the lengthy rehabilitation process. All patients hoped for long-term implementation of this model. A 57-year-old man stated the following: ‘Yes, I hope this becomes a permanent program’.

#### Theme 2: Patient-derived recommendations

1. Enhancing the ePRO model: The patients placed great importance on the timeliness of doctors’ feedback and hoped that doctors would promptly address the information provided by patients in their forms. The relevance of this concern was reflected in system data. They also wished to improve the communication methods and feedback channels between doctors and patients during the management process. Additionally, there was a desire for the management model to be more comprehensive and content rich. A 59-year-old man stated the following: ‘Feedback needs to be prompt—delays could undermine confidence in the process’. A 66-year-old woman stated the following: ‘In my view, the new model should include a bit more content or perhaps provide more guidance for patients in the future’.
2. Improving the ePRO system: A user-friendly interface can enable patients to complete symptom reports more effectively. Easily recognisable app icons can improve the completion rate of patient forms. Additionally, adding sections on the platform for patients to self-report open questions can enhance their engagement. A 72-year-old man stated the following: ‘Adding a free-text section would be ideal’. A 57-year-old man stated the following: ‘I recommend simplifying the verification code’.

## Discussion

Symptom monitoring using ePROs has been widely applied in oncology and perioperative care, where it can improve symptom tracking, patient engagement, and clinical outcomes [8–10]. Our findings are consistent with these, suggesting that the ePRO model could similarly enhance postoperative care by providing timely feedback and supporting better symptom management.

The ePRO model enhances patient-doctor interaction [25], supports symptom management, and maintains a strong connection between patients and doctors after discharge [26]. This continuing care likely reduces anxiety and complications, saving time, energy, and costs [27]. From the perspective of psychological reassurance and actual effectiveness, both aspects benefit patients, potentially explaining their universal satisfaction with the model. However, acceptance was not entirely free of burden. One patient reported that the doctor’s reminders to document symptoms and expressions of concern created a psychological burden for her. She was afraid that the doctor would call to inform her of bad news. This finding indirectly highlights potential psychological obstacles that patients may face during the promotion of the model [28, 29]. Future implementation of this model will require better education of patients (e.g. providing detailed explanations at the time of enrolment) to promote shared decision making and alleviate stress or burden [30].

Our findings should be interpreted not only as evidence of patient acceptance, but also as an indication that the perceived value of ePRO-based symptom management depends on timely clinician feedback, clear patient education, and a user-friendly system design. This interpretation is consistent with recent qualitative studies in thoracic surgery, which have shown that connection with the clinical team, convenience, and self-reflection can motivate ePRO use, whereas unclear purpose, recovery-related symptom burden, and technology barriers may limit engagement [13]. Thus, the value of the ePRO model may lie not simply in digital symptom reporting itself, but in embedding symptom reporting within a responsive postoperative care process.

The patients recommended some improvements to the ePRO model and the form-filling system during the interviews. The positive response from doctors regarding early alerts may be key to the successful management of symptoms through ePROs. Some participants expressed the need for additional guidance when they used the ePRO system, including clearer instructions for form completion and expectations regarding clinical responses. Therefore, future clinical applications of this model must prioritise how doctors respond to early warning signals [13, 31]. Simplifying the questionnaire scales could be another critical factor for the routine application of this model in clinical practice. Overly lengthy scales that require extended completion times may cause patients to develop an aversion to such models, negatively impacting their compliance. On the basis of this trial, our team developed a simplified version of the symptom assessment scale (PSA-Lung), reducing the number of items from 22 to 9 to improve clinical feasibility and patient usability [32].

Although clinician workload was not directly evaluated in this patient interview study, it is likely to be an important issue for the long-term clinical implementation of the ePRO model. Therefore, reasonable allocation of human resources and effective management of working hours to enable timely responses to patient information may be the main solution to this problem. Additionally, robust hardware and software support is essential to ensure a seamless patient experience. A simple, user-friendly, easily recognisable, and responsive form-filling interface may be important in encouraging long-term patient participation.

This study has several limitations. First, conducted in a trial setting with relatively device-proficient participants, this study may not fully reflect routine clinical practice, and the perspectives of patients with lower digital literacy may have been underrepresented. Second, the China-specific context, particularly the use of a WeChat-based platform, may limit transferability to other healthcare systems. Third, all patient interviews in this study were conducted via telephone, which limited the depth and duration of the interviews. Future interviews may consider alternative formats (e.g. in-person or video-based) rather than the telephone-based format to potentially enhance the depth of responses. Fourth, social desirability bias may have contributed to the predominance of positive responses because participants were recruited from a trial conducted by their treating clinical team. Fifth, although the transcripts were manually checked against the original audio, the use of software-assisted transcription may still have introduced subtle shifts in wording or meaning.

## Conclusions

This China-based qualitative interview study showed that patients undergoing lung cancer surgery accepted the ePRO-based symptom management model and valued its support during recovery. Additionally, we obtained actionable suggestions for the ePRO model and ePRO system that may enhance patient engagement and clinical implementation in China.

## Electronic Supplementary Material

Below is the link to the electronic supplementary material.

Supplementary Material 1

Supplementary Material 2

## Data Availability

The anonymized datasets generated and analysed in this study are available from the corresponding author upon reasonable scientific request. Data sharing will be considered for non-commercial research purposes or regulatory applications, contingent upon execution of a formal data access agreement to ensure compliance with ethical and privacy protections.
