# Supplementary material for: Patient perspectives on electronic patient-reported outcome-based symptom management after lung cancer surgery: a qualitative study
Source: J Patient Rep Outcomes. 2026 May 19;10:116. doi: 10.1186/s41687-026-01083-4 (PMC13365064; doi:10.1186/s41687-026-01083-4)
Supplement: Supplementary file 1 — Supplementary Material 1 [file 41687_2026_1083_MOESM1_ESM.docx]

**Semi-Structured Interview Guide**

*Methodology: 10–30-minute telephone interviews conducted with postoperative ePRO participants*

**Interview requirements:**

1. The interview involves 20 patients in the intervention group.
2. Quiet room, one-on-one phone interviews lasting 10–30 minutes + audio recording. There is no need to avoid the presence of family members.
3. Name the audio files as: Patient’s name + interviewer’s name + date of interview.
4. Record the number of patients who declined and the reasons for declining.

**Interview outline:**

**Introduction:**

Hello, my name is _________. I am from the Sichuan Clinical Research Center for Cancer, Sichuan Cancer Hospital. Thank you for participating in our study. Our research focuses on ‘Patient-Reported Outcome-Based Symptom Management After Lung Cancer Surgery’. This study requires you to complete daily and weekly symptom questionnaires online.

Just to confirm, are you participating in the study I described?

- If not, provide other prompts about the study, such as:
  - The questionnaire you regularly complete on the WeChat mini-program.
  - The study you enrolled in back in [date] before your lung surgery.
  - The survey that my colleague, [study staff], calls you about.
- If they are not familiar with the study, the interview was terminated.

I would like to remind you that you agreed to participate in this follow-up interview when you registered for this study. This interview aims to understand your experiences and feelings regarding ‘the electronic patient-reported outcome-based symptom management model you participated in’. The interview will last approximately 10-30 minutes. Are you available to talk now?

**Confidentiality:**

First, our team will keep the content discussed here strictly confidential. This interview will not affect your treatment and care in any way, and I encourage you to speak freely without any concerns.

Please understand that there are no right or wrong answers to these questions. Our main goal is to learn from you and ensure you feel comfortable sharing your experiences.

Before we begin, I would like to state that this conversation will be recorded so that we can remember the key points discussed. You may request that I stop recording at any time, or you may directly indicate your preference not to answer a particular question.

Do you have any questions before we start?

**Experiences and feelings:**

[Turn on the recording device]

Today, I will ask you about the symptom monitoring survey and your feelings regarding your participation in this study.

First, please reflect on your past experience reporting symptoms on the WeChat mini-program.

Questions:

1. Do you think this model (electronic patient-reported outcome-based proactive symptom management) is helpful for your postoperative recovery?

**PROMPT**: What specific help did it provide you, and what problems did it solve for you?

1. Do you think this model is necessary?

**PROMPT**: Why? If you believe it is unnecessary, could you explain your reasons?

1. How satisfied are you with this model?

**PROMPT**: Why? If you are not satisfied, could you specify which aspects you are dissatisfied with?

1. Do you believe this model interferes with your life or increase your burden?

**PROMPT**: Why? If you believe the model interferes with your life or increases your burden, could you provide specific details?

1. What suggestions do you have for this model?

**PROMPT**: In which areas do you believe the model could be improved, such as the way doctors provide feedback and the timeliness of their responses?

1. Are you satisfied with the WeChat mini program/electronic form system?

**PROMPT**: Why? If you are not satisfied, could you specify which aspects you find unsatisfactory?

1. What suggestions do you have for improving the WeChat mini program/electronic form system?

**PROMPT**: Which aspects of the questionnaire system do you believe require improvement, such as font size, interface simplicity, and ease of access?

**Conclusions:**

Thank you!

I sincerely appreciate your time and effort in participating in this interview.

[Turn off the recording device]
